# Annealing-Fabricated *Poria cocos* Glucan-Tannic Acid Composite Hydrogels: Integrated Multifunctionality for Accelerated Wound Healing

**DOI:** 10.3390/gels12010096

**Published:** 2026-01-22

**Authors:** Yong Gao, Ruyan Qian, Chenyi Feng, Dan Li, Xinmiao He, Wengui Xu, Jiaxin Zhu, Zongbao Zhou

**Affiliations:** 1 Hubei Key Laboratory of Resource Utilization and Quality Control of Characteristic Crops, College of Life Science and Technology, Hubei Engineering University, Xiaogan 432000, China; 15971283200@163.com (Y.G.); yangzhouman1105@163.com (R.Q.); m18040509631@163.com (C.F.); 15549157552@163.com (D.L.); 13296693616@163.com (X.H.); m15870787121@163.com (W.X.); 2 School of Architecture, Hubei Engineering University, Xiaogan 432000, China

**Keywords:** *Poria cocos* glucan hydrogel, tannic acid, wound healing

## Abstract

Multifunctional wound dressings integrating moisture retention, antibacterial activity, and bioactive delivery are in demand, yet balancing structural stability and functional synergy in polysaccharide hydrogels remains a challenge. This study focused on developing such advanced dressings. *Poria cocos* glucan (PCG) hydrogels were fabricated via annealing, with PCG-4 (4 wt.%) identified as the optimal matrix. PCG-tannic acid (TA) composite hydrogels were subsequently prepared via TA loading, followed by systematic property characterization and in vivo wound healing evaluation in a rat full-thickness wound model. The composite hydrogel exhibited balanced porosity (56.7 ± 3.4%) and swelling (705.5 ± 11.3%), along with enhanced mechanical rigidity. It enabled temperature-responsive TA release, coupled with high antioxidant activity and antibacterial efficacy. Additionally, it showed excellent biocompatibility (hemolysis rate <2%; NIH-3T3 cell viability >98%) and accelerated rat wound closure with enhanced collagen deposition, suggesting a beneficial combined effect of the composite’s components. PCG-TA holds promise as an advanced wound dressing, and the scalable annealing fabrication strategy supports its translational application potential.

## 1. Introduction

Wound healing is a sophisticated, multi-stage physiological cascade involving inflammation, proliferation, and tissue remodeling, and its dysregulation gives rise to a formidable global healthcare challenge [1]. Chronic wounds such as diabetic foot ulcers and pressure sores affect millions of individuals annually, with persistent infections, excessive oxidative stress (ROS), and impaired extracellular matrix (ECM) remodeling often leading to severe complications, including tissue necrosis, limb amputation, and even life-threatening sepsis [2]. These pathological conditions not only deteriorate patients’ quality of life but also impose substantial economic burdens on healthcare systems worldwide. Traditional wound dressings, including gauze and hydrocolloid films, merely serve as passive physical barriers to prevent external contamination; they lack the capacity to maintain a moist microenvironment critical for epithelialization, inhibit pathogenic bacteria such as *Escherichia coli* (*E. coli*) and *Staphylococcus aureus* (*S. aureus*), or scavenge ROS that disrupt fibroblast activity and collagen synthesis [3]. This functional deficiency underscores an urgent clinical demand for multifunctional wound dressings that integrate structural stability, moisture retention, antibacterial activity, antioxidant capacity, and biocompatibility in a single platform [4]. Polysaccharide-based hydrogels have emerged as promising candidates given their ECM-mimicking structure and inherent biocompatibility, yet most existing formulations suffer from limited functional synergy and insufficient mechanical robustness, highlighting the need for novel strategies to fabricate advanced hydrogel dressings with integrated therapeutic functions.

Among natural polysaccharides, *Poria cocos* glucan (PCG), a linear β-glucan derivative, has attracted growing attention as a wound dressing matrix due to its excellent biocompatibility and ECM-like structure, which favors moisture retention, cell adhesion/proliferation, and tissue repair [5,6]. Recent studies on PCG and its derivatives have demonstrated diverse bioactive potentials: PCG-modified Janus nanofibrous membranes with thickness gradients exhibit superior hemostatic performance and promote epithelial regeneration [7]; PCG hydrogels loaded with natural flavonoids possess remarkable antibacterial and persistent antioxidant capacities [8]; water-insoluble PCG can alleviate antibiotic-associated diarrhea by regulating intestinal microbiota and reducing inflammatory cytokines [9]. Despite these advances, pure PCG still has inherent limitations: insufficient standalone immunostimulatory activity and a lack of antibacterial/antioxidant functions critical for regulating the pathological wound microenvironment. Similar to curdlan, pure PCG’s functional deficiency highlights the necessity of integrating bioactive components to construct a multifunctional platform for optimizing wound healing efficacy.

In previous studies, PCG hydrogels have been fabricated via alkali dissolution-acid precipitation, but this method yields materials with insufficient mechanical strength, which limits their practical application in wound dressings requiring structural stability [10,11]. This constitutes a critical limitation that motivates the innovation of this study: we employed an annealing technique to prepare PCG hydrogels, aiming to enhance mechanical performance. Our research team previously validated the annealing strategy’s superiority in curdlan-based hydrogels, fabricating curdlan-tannic acid (TA) hybrid hydrogels with excellent physicochemical properties, favorable biocompatibility, and multifunctional therapeutic effects. These hydrogels were further confirmed to accelerate in vivo wound healing in full-thickness skin defect models [12]. Therefore, this study aimed not merely to combine PCG with TA, but to evaluate the utility of an annealing-based fabrication strategy in creating a mechanically robust PCG-TA composite hydrogel that integrates essential wound dressing functions—moisture management, controlled antioxidant/antibacterial release, and biocompatibility—within a single, simple processing platform. We hypothesize that this annealing-enabled fabrication can not only significantly enhance mechanical strength but also synergize PCG’s biocompatibility and structural advantages with TA’s antibacterial, antioxidant, and bioactive delivery properties, constructing a multifunctional platform to comprehensively regulate the wound microenvironment and promote efficient wound healing.

To validate this hypothesis, this study fabricated PCG hydrogels with varied concentrations via an annealing strategy, and screened PCG-4 (4 wt.%) as the optimal matrix according to comprehensive performance benchmarks. PCG-TA composite hydrogels were then constructed via TA loading, followed by systematic characterization of their physicochemical, mechanical, and functional properties, including TA release, antioxidant activity, antibacterial efficacy, and biocompatibility. A rat full-thickness wound model was further employed to assess in vivo wound healing efficacy, ultimately verifying the composite hydrogel’s potential as an advanced wound dressing.

## 2. Results and Discussion

### 2.1. Fabrication and Characterization of PCG Annealed Hydrogels

#### 2.1.1. Annealing-Assisted Fabrication and Physicochemical Properties

**Figure 1:**
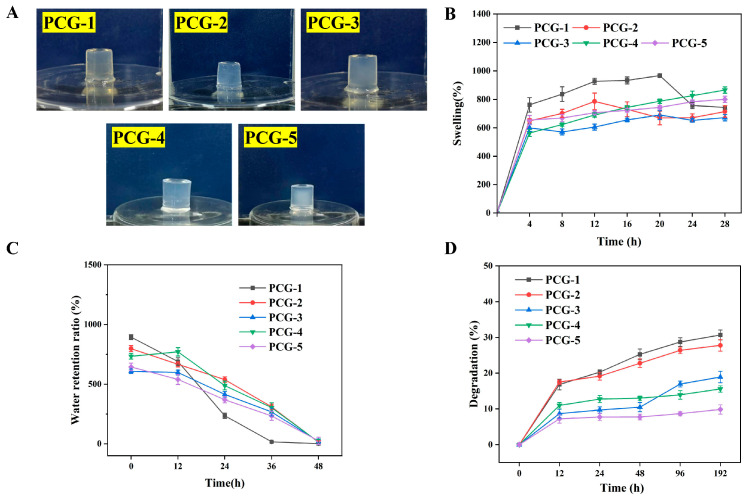
Physicochemical properties of pure PCG hydrogels with different concentrations. (**A**) Macroscopic appearance of PCG hydrogels. (**B**) Swelling ratio curves of PCG hydrogels over time. (**C**) Water retention ratio curves of PCG hydrogels over time. (**D**) In vitro degradation rate curves of PCG hydrogels over time. Data are presented as mean ± standard deviation (n = 3).

The PCG hydrogels (1–5 wt.%, denoted PCG-1 to PCG-5) were fabricated via an annealing technique: this process regulates the molecular motion of β-glucan chains (the core component of PCG), promoting interchain hydrogen bonding and physical entanglement to construct stable 3D networks—here, β-glucan’s linear chain structure enables ordered arrangement under annealing, forming dense entanglement regions that inherently enhance gel structural integrity [13]. All formulations formed self-supporting translucent monoliths (Figure 1A), confirming successful gelation driven by β-glucan chain entanglement. For physicochemical properties: PCG-1 exhibited the highest equilibrium swelling (966.7 ± 12.3% at 24 h) due to low β-glucan chain entanglement density and high network porosity. This swelling capacity is notably higher than that of PCG hydrogels prepared via the conventional method of alkali dissolution followed by pH adjustment-induced gelation (~500%) [14], but it lost water rapidly (declining to 1.03 ± 1.1% at 48 h) as loose networks trap mostly evaporable free water and degraded fastest (28.7 ± 1.4% mass loss at 48 h) via exposed glycosidic bonds; in contrast, PCG-4 showed balanced performance—787.1 ± 16.2% swelling (24 h), 303.4 ± 40.3% water retention (48 h), and 15.4 ± 20.3% degradation (48 h)—a result of moderate β-glucan chain entanglement, which provides sufficient water infiltration space while stabilizing bound water via hydrogen bonding and reducing glycosidic bond exposure [15]. This trend confirms that β-glucan concentration (and thus annealing-induced entanglement) directly modulates the hydrogels’ water handling and stability, with PCG-4 achieving a functional balance absent in overly loose (PCG-1) or dense (PCG-5) formulations.

#### 2.1.2. Morphology and Porosity

**Figure 2:**
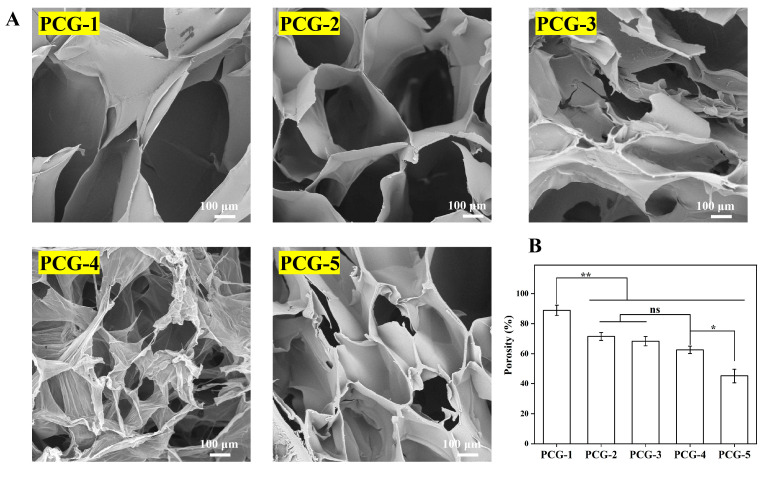
Microstructure and porosity of pure PCG hydrogels. (**A**) Cross-sectional SEM morphologies of PCG hydrogels (scale bar: 100 μm). (**B**) Porosity statistics of PCG hydrogels. Statistical significance: ** *p* < 0.01, * *p* < 0.05, ns = no significant difference. Data are presented as mean ± standard deviation (n = 3).

Figure 2A (SEM micrographs) and Figure 2B (porosity quantification) reveal the structural basis for the above physicochemical trends: PCG-1 displayed a loose 3D network with large pores (porosity: 88.89 ± 3.4%) due to sparse β-glucan chain entanglement, while increasing PCG concentration enhanced annealing-induced chain packing, reducing pore size and thickening pore walls—PCG-4 exhibited a moderately dense network with a porosity of 62.58 ± 2.4% (significantly higher than PCG-5’s 45.12 ± 4.6%, *). Here, β-glucan acts as the network backbone: its chain entanglement (tuned by concentration and annealing) dictates pore structure, and PCG-4’s intermediate porosity directly corresponds to its balanced swelling/retention—providing sufficient water penetration space while maintaining network support, validating the structure-property correlation mediated by β-glucan. Notably, the porosity of PCG-4 (62.58 ± 2.4%) falls within the range (60–90%) generally recognized as favorable for cell migration, nutrient permeation and exudate absorption in wound dressing applications [16], which aligns with the porosity requirements of clinical and reported polysaccharide-based wound dressings.

#### 2.1.3. Rheological and Mechanical Performances

**Figure 3:**
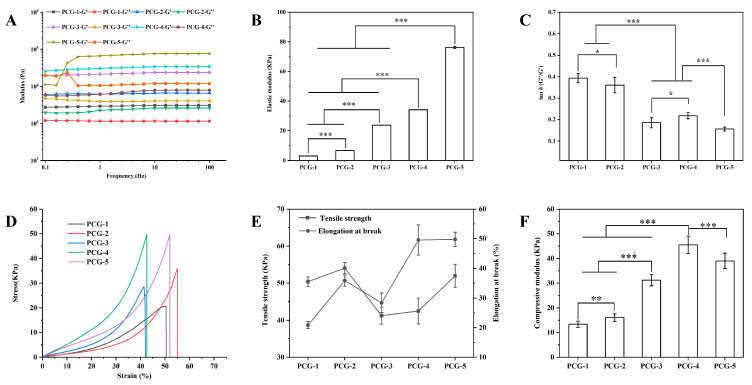
Mechanical properties of pure PCG hydrogels. (**A**) Frequency-dependent storage modulus (G′) and loss modulus (G″) curves of PCG hydrogels. (**B**) Elastic modulus statistics of PCG hydrogels. (**C**) Loss tangent (tanδ) statistics of PCG hydrogels. (**D**) Stress–strain curves of PCG hydrogels. (**E**) Tensile strength and elongation at break statistics of PCG hydrogels. (**F**) Compressive modulus statistics of PCG hydrogels. Statistical significance: * *p* < 0.05, ** *p* < 0.01, *** *p* < 0.001. Data are presented as mean ± standard deviation (n = 3).

Figure 3 characterizes the rheological and mechanical properties of the hydrogels, whose behaviors are rooted in β-glucan’s chain entanglement (enabled by annealing). For rheological properties, all samples exhibited frequency-independent storage modulus (G′, Figure 3A) within the tested frequency range (0.1–10 Hz), confirming stable elastic gel behavior that is critical for maintaining structural integrity during wound dressing application. The elastic modulus (G′, quantified in Figure 3B) showed a distinct concentration-dependent trend: PCG-1 had the lowest G′ (3051.2 ± 24.0 Pa) due to sparse β-glucan chain entanglement, while the modulus increased progressively with PCG concentration, with values for PCG-2, PCG-3, and PCG-4 being 8762.5 ± 45.3 Pa, 19,845.6 ± 62.7 Pa, and 34,173.7 ± 75.84 Pa, respectively. This storage modulus endows PCG-4 with sufficient mechanical strength for practical handling and on-wound application, which is comparable to that of other polysaccharide-based hydrogels designed for wound healing, confirming its applicability as a wound dressing [17]. Notably, PCG-5 exhibited a slight decrease in G′ (42,689.3 ± 81.2 Pa) compared to the increasing trend, which is attributed to overpacking of β-glucan chains during annealing, where excessive entanglement leads to uneven chain distribution and local structural defects that weaken the overall network rigidity [18]. PCG-4’s G′ was 11× higher than PCG-1, reflecting denser chain entanglement that enhances elastic stiffness, while its loss factor (tanδ = 0.218 ± 0.014, Figure 3C) remained <1 (indicative of elastic dominance) and higher than PCG-5 (tanδ = 0.156 ± 0.009). This suggests PCG-4 avoids the brittleness of PCG-5, as moderate tanδ values indicate a balance between elastic rigidity and viscous dissipation—essential for adapting to wound surface deformation without fracture [19].

For mechanical properties, the stress–strain curves (Figure 3D) further reveal the structural-mechanical correlation. PCG-1’s curve exhibited a shallow slope and long elongation plateau (elongation at break: 65.2 ± 4.8%), characteristic of a soft, weak network with insufficient chain entanglement to resist tensile stress. In contrast, PCG-5’s curve showed a steep initial slope (high initial stiffness) but a sharp drop in stress after reaching the yield point (elongation at break: 28.3 ± 2.9%), reflecting brittle fracture caused by over-entangled chains that cannot dissipate stress through chain sliding [20]. PCG-4’s stress–strain curve displayed an optimal balance: a moderate initial slope (indicating sufficient rigidity) followed by a gradual stress increase before fracture, resulting in the highest tensile strength (49.5 ± 5.1 Pa, Figure 3E) and a high elongation at break (42.5 ± 3.5%). This profile confirms PCG-4’s combination of strength and ductility, which is directly attributed to moderate β-glucan chain entanglement that is sufficient to form a robust network (leveraging the inherent rigidity of β-glucan chains) while retaining enough chain mobility to accommodate deformation [21]. Consistent with tensile properties, PCG-4’s compressive modulus (45.5 ± 3.5 kPa, Figure 3F) was the highest among all formulations, while PCG-5’s modulus decreased to 38.99 ± 3.1 kPa due to overpacked chains that generate internal stress concentrations under compression, leading to premature structural failure.

The reason for the property differences lies in the combined regulation of PCG concentration and annealing process on chain entanglement and network structure. Low glucan concentration (PCG-1) leads to sparse chain entanglement during annealing, resulting in large pores, high swelling, but poor water retention and fast degradation. High concentration (PCG-5) causes over-entanglement, forming compact pores that reduce swelling but induce brittleness. Moderate concentration (PCG-4) achieves balanced annealing-induced entanglement, generating intermediate porosity that coordinates water handling, degradation, and mechanical properties [22].

Collectively, PCG-4’s performance (balanced physicochemical properties, moderate porosity, robust rheological/mechanical stability) is a multifunctional outcome of β-glucan’s structural contribution and annealing-induced network regulation, making it the optimal formulation for subsequent bioactivity studies.

### 2.2. Fabrication and Characterization of PCG-TA Composite Hydrogel

**Figure 4:**
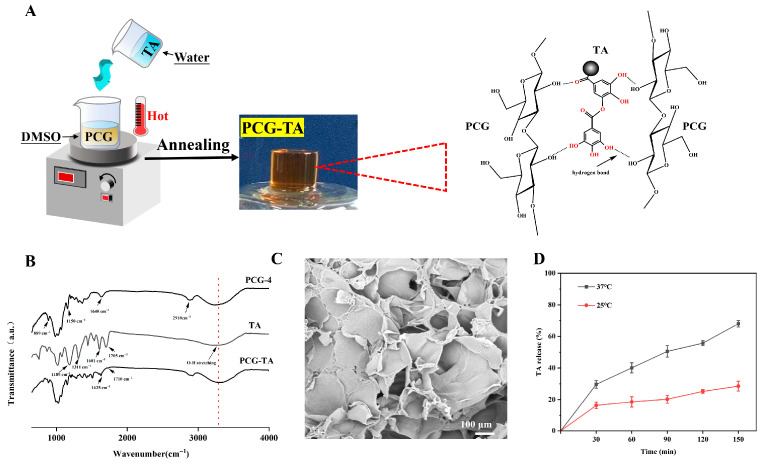
Preparation, microstructure and functional properties of PCG-TA composite hydrogel. (**A**) Left: Schematic illustration of PCG-TA composite hydrogel preparation via annealing; Right: Schematic diagram of hydrogen bonding crosslinking between PCG chains and TA molecules; Inset: Photographic image of the prepared PCG-TA hydrogel. (**B**) FTIR spectra of PCG-4, TA, and PCG-TA composite hydrogel. (**C**) Cross-sectional SEM micrograph of PCG-TA composite hydrogel (scale bar: 100 μm). (**D**) Cumulative release profiles of TA from PCG-TA composite hydrogel at 25 °C and 37 °C (data are presented as mean ± standard deviation, n = 3).

PCG-TA composite hydrogel was fabricated by incorporating tannic acid (TA) into PCG-4 via annealing (Figure 4A): TA interacted with β-glucan chains of PCG-4 via hydrogen bonding during thermal treatment, yielding a self-supporting gel (Figure 4A, inset). The integration of TA and its interaction with the PCG network were confirmed by FTIR spectroscopy (Figure 4B). The spectrum of pure PCG-4 exhibited characteristic bands at approximately 3300 cm^−1^ (O–H stretching), 2910 cm^−1^ (C–H stretching), 1639 cm^−1^ (O–H bending vibration), 1150 cm^−1^, and 889 cm^−1^ (β-glycosidic linkages) [23]. Pure TA showed its distinctive peaks at 1705 cm^−1^ (ester C=O stretch), 1601 cm^−1^ (aromatic C=C stretch), 1311 cm^−1^, and 1189 cm^−1^ (C–O and phenolic O–H vibrations) [24]. In the spectrum of the PCG-TA composite, the carbonyl stretching vibration of TA shifted from 1705 cm^−1^ to 1710 cm^−1^, indicating the participation of the C=O group in hydrogen bonding as an acceptor [25]. More notably, a strong and consolidated band was observed at 1625 cm^−1^ in the composite, which is a superposition of the shifted characteristic peaks of PCG-4 and TA. Specifically, the peak of PCG-4 at ~1639 cm^−1^ shifted to lower wavenumbers, while TA’s aromatic C=C stretch peak at ~1601 cm^−1^ shifted to higher wavenumbers. This mutual shift directly reflects the formation of hydrogen bonds between the hydroxyl groups of PCG and the phenolic hydroxyl/carboxyl groups of TA [26].

Cross-sectional SEM imaging (Figure 4C) further corroborates the formation of the hydrogen-bonded PCG-TA network by visualizing the microstructural alterations induced by TA incorporation. Compared to the moderately dense network of pure PCG-4 (62.6 ± 2.4% porosity), the PCG-TA composite exhibited a denser, more compact 3D network with reduced average pore size, resulting in a lower porosity of 56.7 ± 3.4%. This microstructural densification is a hallmark of successful crosslinking between TA and polysaccharide matrices, as TA acts as a hydrogen-bonding crosslinker to bridge β-glucan chains and fill partial network pores—consistent with observations in other polymer-TA composite hydrogel systems where intermolecular hydrogen bonding induces similar structural consolidation [27]. The SEM results provide direct morphological evidence to complement the FTIR spectral data, collectively validating the formation of a robust hydrogen-bonded PCG-TA network. These spectral changes provide direct molecular-level evidence for the formation of an extensive hydrogen-bonded network between PCG and TA, which is the primary physical crosslinking mechanism responsible for the composite’s enhanced structural integrity. This network of dynamic, yet robust, hydrogen bonds not only reinforces the matrix but also directly contributes to the observed macroscopic property enhancements. It underpins the significant increases in mechanical moduli and the improved water retention, while preserving the essential flexibility required for a functional wound dressing [28].

The key properties of PCG-TA were summarized in Table 1. PCG-TA exhibited a reduced porosity (56.7 ± 3.4% vs. PCG-4’s 62.6 ± 2.4%), as TA filled partial network pores via β-glucan-TA hydrogen bonding. This structural change lowered 24 h swelling (705.5 ± 11.3% vs. 787.1 ± 16.2%) by restricting water infiltration, but enhanced 48 h water retention (368.9 ± 10.3% vs. 303.4 ± 40.3%)—TA introduced additional hydrogen bond sites to stabilize bound water, reducing evaporative loss. 48 h non-enzymatic degradation (31.1 ± 9.1% vs. 15.4 ± 20.3%) accelerated moderately, as TA weakened local β-glucan chain entanglement, increasing glycosidic bond accessibility to water [29].

TA’s crosslinking effect elevated PCG-TA’s elastic modulus to 61,221.1 ± 65.6 Pa, a value significantly higher than that of a similar TA-polysaccharide composite gel (~10,000 Pa) [30], while also increasing the compressive modulus (66.3 ± 6.5 kPa vs. 45.5 ± 3.5 kPa) and tensile strength (59.6 ± 3.3 kPa vs. 49.5 ± 5.1 kPa), thereby reinforcing network rigidity. Notably, tanδ (0.211 ± 0.011 vs. 0.218 ± 0.014) remained nearly unchanged, preserving the gel’s elastic flexibility—this balance of strength and ductility is critical for bioactive applications requiring structural integrity and deformability.

### 2.3. TA Release Behavior

PCG-TA exhibited temperature-dependent TA release (Figure 4D): at 37 °C (physiological temperature), TA release reached 68.4 ± 2.1% at 150 min, while only 28.5 ± 3.1% was released at 25 °C. This trend directly correlated with PCG-TA’s swelling behavior: 37 °C promoted greater network expansion (~90% swelling at 150 min), widening diffusion channels for TA; in contrast, 25 °C restricted swelling, maintaining a denser network that hindered TA diffusion [31]. The 150 min duration targets early-stage antioxidant action critical for acute wound care [32]. Although the release did not reach equilibrium, this early active phase dominates therapeutic efficacy for initial oxidative stress relief in acute wounds. Notably, the initial TA release observed here is closely associated with the non-enzymatic degradation behavior of PCG-TA. The previously reported significant increase in mass loss (from ~15% for pure PCG to ~30% for PCG-TA) is primarily attributed to the dissolution and diffusion of unbound or loosely bound TA molecules from the hydrogel matrix into the release medium, which contributes substantially to both the measured weight loss and the early-stage TA release. A minor fraction of the mass loss may also stem from slightly enhanced PCG chain accessibility induced by TA incorporation, rather than extensive enzymatic hydrolysis. This phenomenon, which refers to initial weight reduction and corresponding bioactive release driven by the leaching of unbound components, is consistent with findings in a TA-loaded polysaccharide hydrogel system [33]. This temperature responsiveness is functionally relevant: in in vivo scenarios (37 °C), the gel can efficiently release TA to exert bioactivity; during storage (25 °C), slow release minimizes TA loss, extending the gel’s shelf-life. The coupling of release behavior with swelling avoids the need for exogenous stimuli, simplifying practical application.

### 2.4. Antioxidant Activity

**Figure 5:**
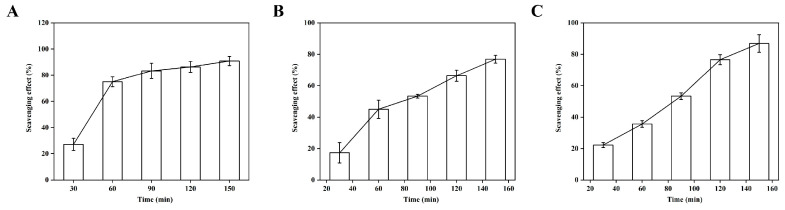
Time-dependent scavenging effects of PCG-TA composite hydrogel on different reactive oxygen species. (**A**) DPPH radical scavenging rate; (**B**) Superoxide anion radical scavenging rate; (**C**) Hydroxyl radical scavenging rate. All data are presented as mean ± standard deviation (n = 3).

To comprehensively assess the ROS-scavenging capacity of PCG-TA hydrogel, we evaluated its activity against DPPH, superoxide anion, and hydroxyl radical in a time-dependent manner, with results tightly coupled to TA release kinetics (Figure 5): DPPH scavenging increased from 27.27 ± 4.6% (30 min) to 90.91 ± 3.5% (150 min), superoxide anion from 17.27 ± 6.5% to 76.91 ± 2.5%, and hydroxyl radical from 22.27 ± 1.5% to 86.9 ± 5.6%. This consistency confirms TA’s polyphenolic groups retain free radical-neutralizing activity post-loading, as hydrogen bonding with PCG avoids structural denaturation of TA’s active sites [34]. The differential scavenging efficiency (DPPH > hydroxyl radical > superoxide anion) arises from ROS reactivity and TA’s binding affinity—hydroxyl radical’s high reactivity leads to rapid quenching, while superoxide anion’s anionic nature weakens interaction with TA’s phenolic hydroxyls, yet the final rates remain biologically effective [35]. Mechanistically, annealing-induced β-glucan chain entanglement constructs a porous, stable matrix that regulates TA’s sustained release via dynamic hydrogen bond dissociation, resolving the bottleneck of rapid bioactive leaching in conventional hydrogels.

### 2.5. Antibacterial Activity

**Figure 6:**
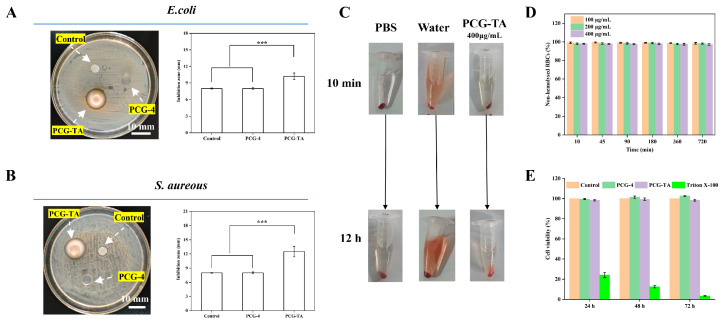
Antibacterial activity, hemocompatibility and cytotoxicity of PCG-4 and PCG-TA hydrogels. (**A**) Antibacterial results against *E. coli*: Photographs of inhibition zones (**left**) and statistical analysis of inhibition zone diameters (**right**) for Control, PCG-4 and PCG-TA groups. (**B**) Antibacterial results against *S. aureus*: Photographs of inhibition zones (**left**) and statistical analysis of inhibition zone diameters (**right**) for Control, PCG-4 and PCG-TA groups. (**C**) Visual photographs of hemolysis test samples (PBS, Water, PCG-TA) at 10 min and 12 h. (**D**) Non-hemolyzed RBC rate curves of PCG-TA hydrogel at different concentrations (100, 200, 400 μg/mL) over time. (**E**) Cell viability of NIH-3T3 cells cultured with Control, PCG-4, PCG-TA and Triton X-100 groups at 24, 48 and 72 h. Statistical significance: *** *p* < 0.001. Data are presented as mean ± standard deviation (n = 3).

The antibacterial performance of PCG-TA was evaluated against Gram-negative *E. coli* and Gram-positive *S. aureus* via disk diffusion assays, as shown in Figure 6A,B, with 5 mm blank disks serving as the control. For *E. coli*, the inhibition zone of both the control and PCG-4 measured approximately 5.0 mm, indicating no significant antibacterial effect; in contrast, PCG-TA exhibited an enlarged inhibition zone of 10.8 ± 0.6 mm. For *S. aureus*, the control and PCG-4 also showed inhibition zones of ~5.0 mm, but PCG-TA’s zone increased to 12.5 ± 1.1 mm, with a statistically significant difference.

This activity enhancement arises from TA’s polyphenolic groups, which interact with bacterial cell membrane components—such as lipopolysaccharides in *E. coli* and peptidoglycan in *S. aureus*—to disrupt membrane integrity and exert bacteriostatic effects [36]. Pristine PCG-4, composed of β-glucan without such functional groups, shows no obvious antibacterial activity, confirming that TA loading endows PCG-TA with targeted antibacterial capacity. The stronger effect against *S. aureus* likely stems from TA’s higher affinity for the thick peptidoglycan layer characteristic of Gram-positive bacteria [37].

### 2.6. Hemocompatibility

Hemocompatibility was assessed via hemolysis rate measurements, as shown in Figure 6C,D. For PCG-TA at concentrations of 100 to 400 μg/mL, the non-hemolyzed red blood cell rate remained above 97% across all time points (10 to 720 min), resulting in a hemolysis rate below 2%—a value well below the 5% threshold defined for biocompatible materials. In contrast, the water control (as observed in Figure 5C) exhibited significant hemolysis, evident from its red supernatant, while PCG-TA maintained a clear supernatant with no obvious hemolysis.

This low hemolysis rate reflects the biocompatible nature of the composite system: PCG is a natural polysaccharide with inherent blood compatibility, and TA interacts mildly with red blood cells without disrupting their membranes. This excellent hemocompatibility aligns with the reported performance of TA-loaded polysaccharide hydrogels for wound healing applications [38]. These results confirm PCG-TA has excellent hemocompatibility, making it suitable for applications involving direct blood contact, such as wound dressings.

### 2.7. Cytotoxicity

Cytotoxicity against NIH-3T3 cells was evaluated via cell viability assays, as presented in Figure 6E. Over 24 to 72 h of incubation, both PCG-4 and PCG-TA maintained cell viability above 98%, with levels comparable to those of the control group. By contrast, the toxic control Triton X-100 reduced cell viability to less than 25% after 24 h and less than 5% after 72 h.

The high cell viability aligns with the biocompatibility of the composite system: PCG is non-toxic to mammalian cells, and TA at the loaded concentration does not induce cytotoxicity. This outcome, paired with the low hemolysis rate and structural stability characterized earlier, confirms PCG-TA is biocompatible and well-suited for in vitro and in vivo bioactive applications.

### 2.8. Wound Healing Phenotype Analysis

**Figure 7:**
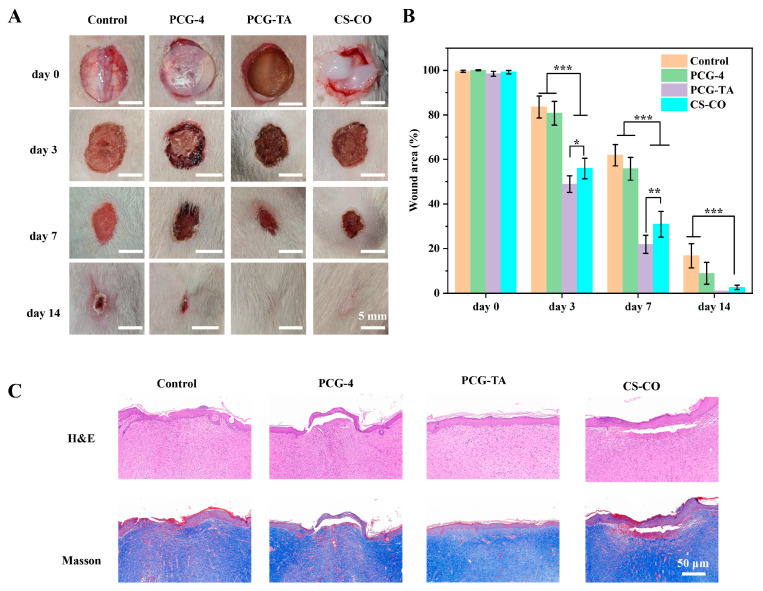
Wound healing evaluation of different groups in rat full-thickness dorsal skin defect model (groups: Control, PCG-4, PCG-TA, commercial chitosan gel ointment (CS-CO)). (**A**) Gross appearance of wounds in each group at day 0, 3, 7 and 14 (scale bar: 5 mm). (**B**) Statistical curves of wound area percentage (relative to day 0) at day 0, 3, 7 and 14. (**C**) H&E staining (top row, evaluating epithelialization and tissue structure) and Masson trichrome staining (bottom row, evaluating collagen deposition) of wound tissues in each group at day 14 (scale bar: 50 μm). Statistical significance: * *p* < 0.05, ** *p* < 0.01, *** *p* < 0.001. Data are presented as mean ± standard deviation (n = 4 per group).

The in vivo wound healing efficacy of PCG-TA was evaluated via murine full-thickness wound models, with control (untreated), PCG-4, and CS-CO (positive control) as references (Figure 7A,B). At day 0, all groups exhibited wound areas close to 100%, showing no significant differences. By day 3, the PCG-TA group showed a wound area of 48.9 ± 3.7%, which was significantly smaller than that of the control (83.6 ± 4.9%) and PCG-4 (80.8 ± 5.3%) groups, indicating accelerated early-stage healing. On day 7, the PCG-TA group’s wound area further reduced to 21.9 ± 4.1%, far lower than the control (61.9 ± 4.8%) and PCG-4 (55.8 ± 5.1%) groups. By day 14, the PCG-TA group achieved nearly complete wound closure (area ratio ~1%), which was significantly superior to the control (16.8 ± 5.4%), PCG-4 (8.9 ± 4.9%), and CS-CO (2.6 ± 1.1%) groups. The nearly complete wound closure (~99%) achieved by day 14 compares favorably with, and in some cases exceeds, the healing rates reported for the advanced hydrogel dressing in comparable full-thickness wound models [39]. This robust healing performance arises from PCG-TA’s multifunctional functions: PCG-4 provides moisture retention and structural support to maintain a favorable healing microenvironment, while TA’s antibacterial and antioxidant activities alleviate inflammation and promote tissue repair.

### 2.9. Histological Evaluation

Histological analysis further validated PCG-TA’s healing-promoting effects. H&E staining (day 14) showed that the control group retained abundant inflammatory cells, with an incomplete epidermal layer and disorganized dermal structure. The PCG-4 group exhibited reduced inflammation but insufficient epidermal repair. In contrast, the PCG-TA group displayed a continuous, intact epidermal layer, markedly reduced inflammatory cell infiltration, and more mature granulation tissue formation in the dermis; the CS-CO group showed partial repair but less consistent epidermal continuity than PCG-TA.

Masson staining revealed that the PCG-TA group had richer, more densely arranged collagen deposition in the wound area. The Control group showed sparse, scattered collagen, while the PCG-4 group had moderate collagen content but a looser arrangement. Abundant collagen deposition indicates that PCG-TA promotes extracellular matrix remodeling—this is attributed to TA’s antioxidant activity (which mitigates oxidative stress-induced damage to fibroblasts) and PCG’s biocompatibility (which supports fibroblast proliferation and collagen synthesis). This accelerated healing outcome is consistent with findings in TA-containing hydrogel systems, where TA acts in conjunction with polysaccharides to enhance tissue repair [40]. Together, these results confirm that PCG-TA accelerates both gross wound closure and histological tissue repair, outperforming PCG-4 and the positive control CS-CO.

These results, combined with PCG-TA’s high antioxidant activity and antibacterial efficacy, indirectly support the potential integrated effect of PCG (moisture retention, cell support) and TA (ROS scavenging, infection inhibition) in reducing inflammation and promoting repair. Follow-up experiments (e.g., detection of inflammatory cytokines and collagen-related gene expression) will further verify the specific mechanism.

## 3. Conclusions

In this study, *Poria cocos* glucan (PCG) hydrogels with tunable properties were successfully fabricated via a simple annealing strategy, and the PCG-4 formulation (4 wt.%) was identified as the optimal matrix due to its balanced porosity, swelling, water retention, mechanical stability, and degradation behavior—all mediated by moderate β-glucan chain entanglement induced by annealing. By incorporating tannic acid (TA) into PCG-4, the PCG-TA composite hydrogel exhibited enhanced structural and functional performance: TA-mediated crosslinking improved mechanical rigidity (elastic modulus: 61,221.1 ± 65.6 Pa; compressive modulus: 66.3 ± 6.5 kPa) while preserving elastic flexibility, and enabled temperature-responsive TA release (68.4 ± 2.1% at 37 °C/150 min) that retained high antioxidant activity (DPPH scavenging: 90.9 ± 3.5% at 150 min). PCG-TA also demonstrated targeted antibacterial activity and excellent biocompatibility (hemolysis rate <2%; NIH-3T3 cell viability >98%). In vivo, PCG-TA accelerated rat full-thickness wound healing: it reduced the wound area to ~1% by day 14, promoted epidermal continuity, reduced inflammation, and enhanced collagen deposition, outperforming pristine PCG-4 and the positive control CS-CO. Collectively, this work demonstrates that annealing-enabled PCG-TA hydrogels integrate structural support, moisture retention, controlled bioactive release, antibacterial activity, and biocompatibility via integrated interactions between β-glucan and TA. These multifunctional properties highlight PCG-TA as a promising candidate for bioactive wound dressings and other biomedical applications, while the simple annealing fabrication strategy offers scalability for translational use.

## 4. Materials and Methods

### 4.1. Materials

*Poria cocos* glucan (PCG, purity ≥ 95%, weight-average molecular weight: 8–10 × 10^4^ g/mol, monosaccharide composition: ~95% glucose) was purchased from Shanghai Winherb Medical Technology Co., Ltd. (Shanghai, China). Tannic acid (TA, purity ≥ 98%), dimethyl sulfoxide (DMSO, analytical grade) and other chemical reagents were purchased from Shanghai Macklin Biochemical Co., Ltd. (Shanghai, China). Biological reagents, including Dulbecco’s Modified Eagle Medium (DMEM), fetal bovine serum (FBS), penicillin-streptomycin solution, and Cell Counting Kit-8 (CCK-8), were obtained from Solarbio Science & Technology Co., Ltd. (Beijing, China). Healthy male Sprague-Dawley (SD) rats (weight: 200–220 g) were purchased from Shulaibao Biotechnology Co., Ltd. (Wuhan, China). All reagents were used without further purification unless otherwise specified.

### 4.2. Preparation of PCG Hydrogels and PCG-TA Composite Hydrogel

PCG hydrogels and PCG-TA composite hydrogel were fabricated via an annealing technique with modification of our previously reported method [12].

#### 4.2.1. Preparation of Pure PCG Hydrogels

Briefly, PCG was dissolved in DMSO to prepare PCG/DMSO solutions with five different concentrations (1, 2, 3, 4, and 5 wt.%). Each solution was vigorously stirred at 100 °C for 5 min to ensure complete dissolution, and then poured into a polytetrafluoroethylene (PTFE) mold. The mold was transferred to a foam box for annealing treatment, allowing the temperature to cool slowly from 100 °C to 25 °C and be maintained at 25 °C for 12 h to form pure PCG hydrogels. Subsequently, the hydrogels were immersed in distilled water at 4 °C for 48 h, with the distilled water refreshed every 12 h to thoroughly remove residual DMSO. Finally, the hydrogels were freeze-dried at −50 °C for 24 h to obtain dehydrated PCG scaffolds, which were stored in a dry environment for subsequent use. The five pure PCG hydrogels were labeled as PCG-1, PCG-2, PCG-3, PCG-4, and PCG-5, corresponding to PCG concentrations of 1, 2, 3, 4, and 5 wt.%, respectively. Based on comprehensive performance screening, the 4 wt.% PCG hydrogel (PCG-4) was selected as the optimal matrix for the preparation of subsequent composite hydrogels.

#### 4.2.2. Preparation of PCG-TA Composite Hydrogel

Using the optimal PCG-4 matrix as the base, a PCG-TA composite hydrogel with a PCG/TA weight ratio of 1:1 was prepared. Firstly, 10 mL of 4 wt.% PCG/DMSO solution (prepared as described in Section 4.2.1) was heated to 100 °C under vigorous stirring. Then, 10 mL of 0.1 g/mL TA aqueous solution was added dropwise into the PCG/DMSO solution, and the mixture was continuously stirred at 100 °C for 5 min to form a homogeneous system. The mixture was poured into a PTFE mold and annealed in a foam box for 12 h (cooling from 100 °C to 25 °C) to form the PCG-TA composite hydrogel. After annealing, the composite hydrogel was washed with distilled water at 4 °C for 48 h (distilled water refreshed every 12 h) to remove residual DMSO and unbound TA. It was then freeze-dried at −50 °C for 24 h to obtain a dehydrated PCG-TA composite scaffold (labeled as PCG-TA), which was stored in a dry environment for subsequent experiments.

### 4.3. Characterization and Performance Tests [22]

#### 4.3.1. Swelling Ratio Test

The swelling behavior of PCG and PCG-TA hydrogels was evaluated using the gravimetric method. Freeze-dried samples were accurately weighed to obtain the initial dry weight (Wd) and then immersed in phosphate-buffered saline (PBS, pH 7.4) at 37 °C. At predetermined time intervals (4, 8, 12, 16, 20, 24, and 28 h), the samples were taken out, and surface water was gently blotted with filter paper before weighing to obtain the swollen weight (Ws). Each sample was tested in triplicate to ensure data reliability. The swelling ratio was calculated using the following Equation (1):

Swelling (%) = [(Ws − Wd)/Wd] × 100%,(1)

where Ws is the weight of the swollen hydrogel, and Wd is the weight of the freeze-dried hydrogel.

#### 4.3.2. Water Retention Capacity Test

The fully swollen hydrogels were weighed to obtain the saturated water weight (Wsw). The samples were then placed in a constant temperature and humidity chamber (37 °C, 50% relative humidity) to simulate the evaporative environment of the wound surface. The weights of the hydrogels (Wr) were recorded at 12, 24, 36, and 48 h. Three parallel samples were set for each group. The water retention rate (WR) was calculated as Equation (2):

WR (%) = [(Wr − Wd)/(Ww − Wd)] × 100%,(2)

where Wd is the initial dry weight of the hydrogel, and Ww is the saturated water weight of the hydrogel.

#### 4.3.3. In Vitro Degradation Test

The in vitro degradation behavior of the hydrogels was investigated in PBS (pH 7.4) at 37 °C with constant shaking (100 rpm). Freeze-dried samples were weighed to obtain the initial dry weight (W0) and then immersed in 20 mL of the degradation medium. At 12, 24, 48, 96, and 192 h, the samples were retrieved, rinsed thoroughly with distilled water to remove residual degradation medium, freeze-dried again, and weighed to obtain the residual weight (Wt). Each group was tested in triplicate. The degradation rate (DR) was calculated using the following Equation (3):

DR (%) = [(W0 − Wt)/W0] × 100%,(3)

#### 4.3.4. Scanning Electron Microscopy (SEM) Observation

The microstructure of freeze-dried PCG-4 and PCG-TA hydrogel scaffolds was observed by SEM (SU8010, Hitachi, Japan). Before observation, the samples were cut into small pieces (5 mm × 5 mm × 2 mm) and sputter-coated with a thin layer of gold to improve electrical conductivity. The observation was performed under an accelerating voltage of 5 kV, and images of the surface and cross-section of the scaffolds were captured to analyze the pore morphology and distribution.

#### 4.3.5. Porosity Measurement

The porosity of the freeze-dried hydrogel scaffolds was determined by the liquid displacement method using ethanol as the displacement medium. First, the volume of the measuring cylinder was recorded (V1), and then a certain volume of ethanol (V2) was added. The freeze-dried sample was accurately weighed (Wd) and immersed in ethanol, followed by vacuum degassing for 30 min to ensure that ethanol fully filled the pores of the scaffold. The total volume of the system was recorded (V3) after the sample was completely immersed. The porosity (P) was calculated using the following Equation (4):

Swelling (%) = [(Ws − Wd)/Wd] × 100%,(4)

where Vsample is the apparent volume of the freeze-dried hydrogel scaffold, calculated by the geometric dimension method; V1 is the initial volume of the measuring cylinder, V2 is the volume of ethanol added, and V3 is the total volume of ethanol and the sample after immersion.

#### 4.3.6. Rheological Property Test

Rheological properties of the swollen PCG-4 and PCG-TA hydrogels were tested using a rotational rheometer (MCR 302, Anton Paar, Austria) with a parallel plate fixture (diameter = 25 mm, gap = 1 mm). All tests were performed at 37 °C. First, a strain sweep test (strain range: 0.1–100%, frequency = 1 Hz) was conducted to determine the linear viscoelastic region (LVR) of the hydrogels. Then, a frequency sweep test was carried out within the LVR (strain = 1%, frequency range: 0.1–10 Hz) to record the storage modulus (G′) and loss modulus (G″). The loss tangent (tanδ = G″/G′) was calculated to evaluate the viscoelastic balance of the hydrogels. Each sample was tested in duplicate.

#### 4.3.7. Compression Mechanical Test

The compression mechanical properties of the freeze-dried PCG-4 and PCG-TA hydrogels were measured using a universal testing machine (Instron 5967, Norwood, MA, USA). The samples were cut into cylindrical shapes with a diameter of 8 mm and a height of 5 mm. The compression test was performed at a constant loading rate of 1 mm/min until the gel could no longer bear the force and the stress dropped sharply, or the compression strain reached 80% (whichever came first). The compression modulus (Young’s modulus) was calculated from the linear elastic region (strain = 5–10%) of the stress–strain curve. Each group was tested with 5 parallel samples to ensure statistical significance.

#### 4.3.8. FTIR Spectroscopy

FTIR spectra of PCG-4, pure TA, and PCG-TA composite hydrogel were collected using a FTIR spectrometer (Thermo Fisher Scientific, Waltham, MA, USA) in the wavenumber range of 4000–500 cm^−1^. The resolution was set to 4 cm^−1^, and each sample was scanned 32 times to enhance the signal-to-noise ratio. Prior to testing, all samples were ground into fine powders and uniformly mixed with anhydrous KBr (sample-to-KBr mass ratio = 1:100), then pressed into transparent pellets under vacuum.

### 4.4. TA Release Rate Test

The in vitro TA release behavior of the PCG-TA hydrogel was investigated using the dialysis bag method at two temperatures (25 °C and 37 °C) with PBS (pH 7.4) as the release medium [12]. Freeze-dried PCG-TA hydrogel (20 mg) was accurately weighed and placed into a dialysis bag (molecular weight cutoff: 3500 Da). The dialysis bag was sealed and immersed in 50 mL of PBS release medium, followed by incubation in a constant temperature shaker (100 rpm). At predetermined time intervals (0.5, 1, 2, 4, 8, 12, 24, 48, 72 h), 2 mL of the release medium was collected for testing, and an equal volume of fresh PBS was immediately supplemented to maintain a constant total volume. The concentration of TA in the collected solution was determined by a UV-Vis spectrophotometer (UV-2600, Shimadzu, Japan) at the maximum absorption wavelength of TA (276 nm) using a pre-established standard curve of TA concentration vs. absorbance. Each group was set with 3 parallel samples. The cumulative release rate (CR) of TA was calculated using the following Equation (5):

CR (%) = [(Ct × Vt + ΣCi × Vi)/M0] × 100%,(5)

where Ct is the TA concentration in the release medium at time t, Vt is the total volume of the release medium (50 mL), Ci is the TA concentration in the i-th collected solution, Vi is the volume of the collected solution (2 mL), and M0 is the total mass of TA loaded in the PCG-TA hydrogel.

### 4.5. Antioxidant Activity Evaluation

The antioxidant activity of PCG-4 and PCG-TA hydrogels was evaluated via three methods targeting different ROS [41], using a unified sample preparation protocol: freeze-dried hydrogel samples were ground into powder, and a 400 μg/mL sample solution was prepared with distilled water.

#### 4.5.1. DPPH Radical Scavenging Rate

2 mL of sample solution was mixed with 2 mL of 0.1 mmol/L DPPH ethanol solution, shaken thoroughly, and incubated in the dark at room temperature for 30 min. After centrifugation (5000 rpm, 5 min), the absorbance of the supernatant was measured at 517 nm. The scavenging rate (SR) was calculated as Equation (6):

SR (%) = [1 − (A_s_ − A_1_)/A_0_] × 100%, (6)

where A_s_ is the absorbance of the sample-DPPH mixture, A_1_ is the absorbance of the sample control, and A_0_ is the absorbance of the blank control.

#### 4.5.2. Superoxide Anion Radical Scavenging Rate

2 mL of sample solution was mixed with 2 mL of 50 mmol/L Tris-HCl buffer (pH 8.2), incubated at 25 °C for 10 min, and then 0.4 mL of 25 mmol/L pyrogallol solution was added. Absorbance was measured at 320 nm immediately (A_1_) and 4 min later (A_1_′). The scavenging rate was calculated as Equation (7):

SR (%) = [1 − (A_1_′ − A_1_)/(A_0_′ − A_0_)] × 100%, (7)

where A_0_ is the absorbance of the blank group at 0 min; A_0_′ is the absorbance of the blank group at 4 min.

#### 4.5.3. Hydroxyl Radical Scavenging Rate

1 mL of sample solution was mixed with 1 mL of 9 mmol/L FeSO_4_, 1 mL of 9 mmol/L salicylic acid-ethanol, and 1 mL of 8.8 mmol/L H_2_O_2_, then incubated at 37 °C for 30 min. Absorbance was measured at 510 nm (A_s_). The blank group used distilled water instead of the sample (A_b_), and the control group used distilled water instead of H_2_O_2_ (A_0_). Each group was tested in triplicate. The scavenging rate was calculated as Equation (8):

SR (%) = [1 − (A_s_ − A_0_) /A_b_] × 100%, (8)

### 4.6. Antibacterial Activity Test

The antibacterial activity of PCG-4 and PCG-TA hydrogels was evaluated against two common wound pathogenic bacteria: *Escherichia coli* (*E. coli*, ATCC 25922) and *Staphylococcus aureus* (*S. aureus*, ATCC 25923). The test was performed using the disk diffusion method. First, the bacterial strains were activated in Luria–Bertani (LB) liquid medium at 37 °C with shaking (180 rpm) for 12 h, and the bacterial concentration was adjusted to 1 × 10^6^ CFU/mL using sterile PBS. Then, 100 μL of the bacterial suspension was evenly spread on the surface of the LB solid medium plates. The hydrogels were cut into circular disks (diameter = 5 mm) and sterilized by ultraviolet irradiation for 30 min. The sterilized hydrogel disks were placed on the inoculated LB plates, which were then incubated at 37 °C for 24 h. After incubation, the diameter of the inhibition zone around each disk was measured using a vernier caliper. Sterile filter paper disks of the same size were used as the blank control. Each group was set with 3 parallel plates, and the average value of the inhibition zone diameter was calculated.

### 4.7. Hemolysis Test

The hemocompatibility of PCG-4 and PCG-TA hydrogels was evaluated by the in vitro hemolysis test using fresh rat blood [12]. First, 10 mL of fresh rat blood was mixed with 10 mL of sterile PBS, and the mixture was centrifuged at 3000 rpm for 10 min to collect red blood cells (RBCs). The RBCs were washed with sterile PBS 3 times until the supernatant was colorless, and then diluted with sterile PBS to prepare a 2% (*v*/*v*) RBC suspension. Freeze-dried hydrogel samples were ground into powder, and three concentrations of sample solutions (100 μg/mL, 200 μg/mL, 400 μg/mL) were prepared using sterile PBS. Then, 2 mL of each concentration sample solution was added to centrifuge tubes, followed by incubation at 37 °C for 30 min. After that, 2 mL of the 2% RBC suspension was added to each tube and incubated continuously at 37 °C for 60 min. After incubation, the mixture was centrifuged at 3000 rpm for 10 min. The absorbance of the supernatant was measured at 540 nm using a microplate reader. Sterile PBS (2 mL) mixed with 2 mL of 2% RBC suspension was used as the negative control (A negative, hemolysis rate = 0%), and 2 mL of distilled water mixed with 2 mL of 2% RBC suspension was used as the positive control (Ap, hemolysis rate = 100%). Each group was tested in triplicate. The hemolysis rate (HR) was calculated as the following Equation (9):

HR (%) = [(As − An)/(Ap − An)] × 100%,(9)

### 4.8. Cytotoxicity Test on NIH-3T3 Cells

The cytotoxicity of PCG-4 and PCG-TA hydrogels to mouse embryonic fibroblasts (NIH-3T3 cells) was evaluated using the Cell Counting Kit-8 (CCK-8) method. First, freeze-dried hydrogel samples (100 mg) were immersed in 2 mL of DMEM containing 10% FBS and 1% penicillin-streptomycin, and incubated at 37 °C, 5% CO_2_ for 24 h to prepare the sample leaching solution. The leaching solution was filtered through a 0.22 μm sterile filter membrane for sterilization. NIH-3T3 cells were seeded into a 96-well plate at a density of 5 × 10^3^ cells per well and cultured for 24 h to allow cell adhesion. Then, the original medium was discarded, and 100 μL of the sterile sample leaching solution was added to each experimental well; 100 μL of fresh DMEM (containing 10% FBS and 1% penicillin-streptomycin) was added to the blank control wells. The 96-well plate was incubated at 37 °C, 5% CO_2_ for 24, 48, and 72 h, respectively. After each incubation period, 10 μL of CCK-8 reagent was added to each well, and the plate was incubated for another 2 h. The absorbance of each well was measured at 450 nm using a microplate reader. The cell viability (CV) was calculated using the following Equation (10):

CV (%) = (Ae/Ac) × 100%,(10)

where Ae is the absorbance of the experimental wells, and Ac is the absorbance of the blank control wells. Each group was set with 6 parallel wells.

### 4.9. In Vivo Wound Healing Experiment on Rats

Healthy male SD rats (weight: 200–220 g) were housed under a 12 h light/dark cycle, constant temperature (22–25 °C), and constant humidity (50–60%), with free access to food and water. The animal experiment protocol was approved by the Ethics Committee of Hubei Engineering University (protocol code: HEUSK202401010), ensuring strict compliance with the ethical guidelines and procedural frameworks established by the National Research Council’s Guide for the Care and Use of Laboratory Animals.

Before the operation, rats were fasted for 12 h. Rats were anesthetized by intraperitoneal injection of 5% pentobarbital sodium (30 mg/kg body weight). After anesthesia, the dorsal hair of rats was shaved and disinfected with 75% ethanol and iodophor successively. A full-thickness skin defect with a diameter of 10 mm was created on the dorsal midline of each rat using a sterile biopsy punch, ensuring that the dermis and subcutaneous fat layer were completely removed. Hemostasis was achieved by gentle pressure with sterile gauze.

A total of 16 rats were randomly divided into 4 groups (n = 4 per group): blank control group, PCG-4 group, PCG-TA group, and commercial chitosan gel ointment (CS-CO) group. The CS-CO is a locally commonly used clinical wound dressing, selected as a representative reference for preliminary in vivo assessment. The treatment methods for each group were as follows: (1) Blank control group: No dressing was applied to the wound, only sterile gauze was used for loose covering and fixation; (2) PCG-4 group: The freeze-dried PCG-4 hydrogel scaffold (cut into 10 mm diameter disks) was soaked in sterile PBS for 10 min to fully swell, then covered on the wound and fixed with sterile gauze; (3) PCG-TA group: The freeze-dried PCG-TA hydrogel scaffold (10 mm diameter disks) was swollen with sterile PBS and applied to the wound, followed by gauze fixation; (4) CS-CO group: The commercial chitosan gel ointment was evenly applied to the wound surface (dosage: 0.1 g per wound), followed by loose covering and fixation with sterile gauze. The dressings of all groups were changed every 3 days to maintain wound cleanliness, and the wound condition was observed and recorded regularly.

On the 14th day after operation, all rats were anesthetized and sacrificed. The wound tissue (including the wound edge and surrounding normal skin tissue, with a margin of 5 mm) was excised. The collected tissue samples were fixed in 4% paraformaldehyde solution for 24 h, then dehydrated with gradient ethanol, transparentized with xylene, and embedded in paraffin to prepare 5 μm thick serial sections. Hematoxylin–Eosin (HE) staining and Masson trichrome staining were performed on the sections according to the standard kit instructions. The stained sections were observed under an optical microscope (BX53, Olympus, Japan), and images were captured.

## Figures and Tables

**Table 1:** Key properties of PCG-4 and PCG-TA hydrogels.

Property	Porosity	24 h Swelling Ratio	48 h Water Retention Ratio	48 h Non-Enzymatic Degradation Rate	Elastic Modulus	Compressive Modulus	Tensile Strength	tanδ
PCG-4	62.6 ± 2.4%	787.1 ± 16.2%	303.4 ± 40.3%	15.4 ± 20.3%	34,173.7 ± 75.8 Pa	45.5 ± 3.5 kPa	49.5 ± 5.1 kPa	0.218 ± 0.014
PCG-TA	56.7 ± 3.4% *	705.5 ± 11.3% **	368.9 ± 10.3% ***	31.1 ± 9.1% ***	61,221.1 ± 65.6 Pa ***	66.3 ± 6.5 kPa *	59.6 ± 3.3 kPa **	0.211 ± 0.011 *

Notes: Data are presented as mean ± standard deviation (n = 3). Statistical significance (compared with PCG−4): * *p* < 0.05, ** *p* < 0.01, *** *p* < 0.001.

## Data Availability

The data presented in this study are available on request from the corresponding authors. The relevant data are currently being used for ongoing follow-up research and are not publicly available to avoid interfering with the progress of incomplete research projects.

## Abbreviations

The following abbreviations are used in this manuscript:

PCG	*Poria cocos* glucan
TA	Tannic acid
ECM	Extracellular matrix
DMSO	Dimethyl sulfoxide
ROS	Reactive oxygen species
PBS	Phosphate-buffered saline
SEM	Scanning Electron Microscopy
CFU	Colony-forming unit
DMEM	Dulbecco’s Modified Eagle Medium
FBS	Fetal bovine serum
CCK-8	Cell Counting Kit-8
CS-CO	Commercial chitosan gel ointment
*E. coli*	*Escherichia coli*
*S. aureus*	*Staphylococcus aureus*
RBC	Red blood cell
G′	Storage modulus
G″	Loss modulus
tanδ	Loss tangent
HE	Hematoxylin-Eosin

## References

[1] Dong R., Guo B. (2021). Smart wound dressings for wound healing. Nano Today.

[2] Falanga V., Isseroff R.R., Soulika A.M., Romanelli M., Margolis D., Kapp S., Granick M., Harding K. (2022). Chronic wounds. Nat. Rev. Dis. Primers.

[3] Barik S., Keswani K., Ray P., Chakraborty R., Mohini S., Banoth E., Kumar P. (2025). Emerging Smart Biomaterials/Devices to Accelerate Chronic Wound Healing by Modulating Wound Microenvironments. Small.

[4] Alberts A., Bratu A.G., Niculescu A.-G., Grumezescu A.M. (2025). New Perspectives of Hydrogels in Chronic Wound Management. Molecules.

[5] Xu T., Zhang H., Wang S., Xiang Z., Kong H., Xue Q., He M., Yu X., Li Y., Sun D. (2022). A review on the advances in the extraction methods and structure elucidation of Poria cocos polysaccharide and its pharmacological activities and drug carrier applications. Int. J. Biol. Macromol..

[6] Li Z., Sun C., Wang F., Xia Z. (2025). Structural and Gelation Characteristics of Alkali-Soluble β-Glucan from Poria cocos. Gels.

[7] Ding Q., Wu X., Zhou Q., Rui Y., Li H., Zhou Z. (2025). Unidirectional self-pumping esterified Poria cocos polysaccharides Janus nanofibrous membrane for facilitating wound repair. Int. J. Biol. Macromol..

[8] Wang J., Wang Z., Hu J., Meng Y., Zhang B., Zheng G., Wang Q. (2024). Antimicrobial and Antioxidant Hydrogels Based on the Water-Insoluble β-Glucan From Poria cocos Loaded With Total Flavonoids of Ampelopsis grossedentata. ChemistrySelect.

[9] Wan J., Wang F., Xiao Y., Cheng Y., Zheng S., Jiang Q., Tan B., Li X., Chen J., Liao S. (2024). Poria cocos polysaccharide alleviates dextran sulphate sodium-induced ulcerative colitis in mice by modulating intestinal inflammatory responses and microbial dysbiosis. Int. J. Biol. Macromol..

[10] Li W., Fang K., Yuan H., Li D., Li H., Chen Y., Luo X., Zhang L., Ye X. (2023). Acid-induced Poria cocos alkali-soluble polysaccharide hydrogel: Gelation behaviour, characteristics, and potential application in drug delivery. Int. J. Biol. Macromol..

[11] Zhou X., Li Y., Yang Y., Wei L., Wang C., Xu J., Song J., Liu S., Bai J., Suo H. (2025). Regulatory effects of Poria cocos polysaccharides on gut microbiota and metabolites: Evaluation of prebiotic potential. npj Sci. Food.

[12] Zhou Z., Xiao J., Guan S., Geng Z., Zhao R., Gao B. (2022). A hydrogen-bonded antibacterial curdlan-tannic acid hydrogel with an antioxidant and hemostatic function for wound healing. Carbohydr. Polym..

[13] Wu C., Huang J., Chu B., Deng J., Zhang Z., Tang S., Wang X., Wang Z., Wang Y. (2019). Dynamic and Hierarchically Structured Networks with Tissue-like Mechanical Behavior. ACS Nano.

[14] Meng Y., Hu C., Cheng J., Qiu W., Wang Q., Chen X., Chang C., Hu J., Qiu Z., Zheng G. (2023). The extraction, structure characterization and hydrogel construction of a water-insoluble β-glucan from Poria cocos. Carbohydr. Res..

[15] Meng Y., Lyu F., Xu X., Zhang L. (2020). Recent Advances in Chain Conformation and Bioactivities of Triple-Helix Polysaccharides. Biomacromolecules.

[16] Bahlmann L.C., Baker A.E.G., Xue C., Liu S., Meier-Merziger M., Karakas D., Zhu L., Co I., Zhao S., Chin A. (2021). Gelatin-Hyaluronan Click-Crosslinked Cryogels Elucidate Human Macrophage Invasion Behavior. Adv. Funct. Mater..

[17] Xu G., Geng Y., Hu L., Wang J., Pan P., Chen J. (2022). One-Pot Preparation of Polysaccharide-Based Antibacterial Hydrogel for Skin Wound Repair. Macromol. Mater. Eng..

[18] Lin M., Long H., Liang M., Chu B., Ren Z., Zhou P., Wu C., Liu Z., Wang Y. (2021). Antifracture, Antibacterial, and Anti-inflammatory Hydrogels Consisting of Silver-Embedded Curdlan Nanofibrils. ACS Appl. Mater. Interfaces.

[19] Qiao M., Cheng B., Wu W., Liu Y., Wang J., Pei X., Zhu Z., Wan Q. (2025). Elastic sac-shaped hydrogel dressing with responsive antibacterial and pro-healing in movable wounds via MOF activated ink spraying. Biomaterials.

[20] Guo B., Liang Y., Dong R. (2023). Physical dynamic double-network hydrogels as dressings to facilitate tissue repair. Nat. Protoc..

[21] Yamashita I., Yaguchi T., Kobayashi Y., Ito H., Komori H., Noda K., Sato Y., Nishinari K., Nitta Y. (2025). Rheology and NMR studies of true gel formation and gelation mechanism for lentinan. Food Hydrocoll..

[22] Ahmadian Z., Correia A., Hasany M., Figueiredo P., Dobakhti F., Eskandari M.R., Hosseini S.H., Abiri R., Khorshid S., Hirvonen J. (2021). A Hydrogen-Bonded Extracellular Matrix-Mimicking Bactericidal Hydrogel with Radical Scavenging and Hemostatic Function for pH-Responsive Wound Healing Acceleration. Adv. Healthc. Mater..

[23] Ao Y., Hu M., Liu Q., Wang K., Hu C., Shi Z., Meng Y. (2025). The preparation of gel microspheres based on physical crosslinking by β-glucan isolated from Poria cocos residues and the powder characteristics. Carbohydr. Res..

[24] Ma M., Zhong Y., Jiang X. (2020). Thermosensitive and pH-responsive tannin-containing hydroxypropyl chitin hydrogel with long-lasting antibacterial activity for wound healing. Carbohydr. Polym..

[25] Ding Q., Yang C., Xin Y., Huang L., Zhou Q., Zhou Z., Li H. (2025). Qxidized Polysaccharides from Lentinus edodes/Carboxymethyl Chitosan Hydrogel Loaded with Tannic Acid for Accelerating the Infected Wound Repair. ACS Appl. Polym. Mater..

[26] Han Z., Wang M., Hu Z., Wang Y., Tong J., Zhao X., Yue W., Nie G. (2024). Tracking the enzyme-response mechanism of tannic acid-embedded chitosan/γ-polyglutamic acid hydrogel. Commun. Mater..

[27] Yang S., Zhang Y., Wang T., Sun W., Tong Z. (2020). Ultrafast and Programmable Shape Memory Hydrogel of Gelatin Soaked in Tannic Acid Solution. ACS Appl. Mater. Interfaces.

[28] Mo J., Dai Y., Zhang C., Zhou Y., Li W., Song Y., Wu C., Wang Z. (2021). Design of ultra-stretchable, highly adhesive and self-healable hydrogels via tannic acid-enabled dynamic interactions. Mater. Horiz..

[29] Chen S.-K., Liu J.-J., Wang X., Luo H., He W.-W., Song X.-X., Nie S.-P., Yin J.-Y. (2024). Hericium erinaceus β-glucan/tannic acid hydrogels based on physical cross-linking and hydrogen bonding strategies for accelerating wound healing. Int. J. Biol. Macromol..

[30] Lee H.-Y., Hwang C.-H., Kim H.-E., Jeong S.-H. (2018). Enhancement of Bio-Stability and Mechanical Properties of Hyaluronic Acid Hydrogels by Tannic Acid Treatment. Carbohydr. Polym..

[31] Lee J.Y., Shin H.H., Cho C., Ryu J.H. (2024). Effect of Tannic Acid Concentrations on Temperature-Sensitive Sol–Gel Transition and Stability of Tannic Acid/Pluronic F127 Composite Hydrogels. Gels.

[32] Zhang X., Liu K., Qin M., Lan W., Wang L., Liang Z., Li X., Wei Y., Hu Y., Zhao L. (2023). Abundant tannic acid modified gelatin/sodium alginate biocomposite hydrogels with high toughness, antifreezing, antioxidant and antibacterial properties. Carbohydr. Polym..

[33] He X., Liu X., Yang J., Du H., Chai N., Sha Z., Geng M., Zhou X., He C. (2020). Tannic acid-reinforced methacrylated chitosan/methacrylated silk fibroin hydrogels with multifunctionality for accelerating wound healing. Carbohydr. Polym..

[34] Busto F., Scalia A.C., Gentile P., Toniolo S., Cometa S., Liotino S., Cochis A., Mastrorilli P., De Giglio E. (2025). Gellan gum/tannic acid hydrogels for cartilage repair: The versatile role of tannic acid as green crosslinker conferring antibacterial and anti-inflammatory properties. Carbohydr. Polym. Technol. Appl..

[35] Zhang W., Chen Y., Huang J., Xiao Z., Wang F., Zhu G., Liao X., Tang Y., Song Z., Sun J. (2025). Tannic acid: A star molecule in the construction and biomedical applications of hydrogels. Chem. Eng. J..

[36] Sathishkumar G., Gopinath K., Zhang K., Kang E.-T., Xu L., Yu Y. (2022). Recent progress in tannic acid-driven antibacterial/antifouling surface coating strategies. J. Mater. Chem. B.

[37] Hosseini M., Moghaddam L., Barner L., Cometta S., Hutmacher D.W., Medeiros Savi F. (2025). The multifaceted role of tannic acid: From its extraction and structure to antibacterial properties and applications. Prog. Polym. Sci..

[38] Chi C., Li R., Zhao Y., Zhang S., Yang C., Song J. (2025). Antioxidant and biocompatible hydrogel based on genipin-crosslinked quaternized chitosan and tannic acid for skin repair. Int. J. Biol. Macromol..

[39] Cheng M., Hu L., Xu G., Pan P., Liu Q., Zhang Z., He Z., Wang C., Liu M., Chen L. (2023). Tannic acid-based dual-network homogeneous hydrogel with antimicrobial and pro-healing properties for infected wound healing. Colloids Surf. B Biointerfaces.

[40] Han H., Tang L., Li Y., Li Y., Bi M., Wang J., Wang F., Wang L., Mao J. (2023). A multifunctional surgical suture with electroactivity assisted by oligochitosan/gelatin-tannic acid for promoting skin wound healing and controlling scar proliferation. Carbohydr. Polym..

[41] Zhang W., Yang Z.-Y., Cheng X.-W., Tang R.-C., Qiao Y.-F. (2019). Adsorption, Antibacterial and Antioxidant Properties of Tannic Acid on Silk Fiber. Polymers.

